# PiuA promoter mutations confer cefiderocol resistance in *Acinetobacter baumannii*: from *in vitro* evolution to global genomic screening

**DOI:** 10.3389/fmicb.2026.1899246

**Published:** 2026-09-10

**Authors:** Xiaochen Liu, Qingye Xu, Jintao He, Wang Zhang, Sebastian Leptihn, Peng Lan, Yunxing Yang, Yunsong Yu, Xiaoting Hua, Meng Li

**Affiliations:** 1 Department of Clinical Laboratory, Zhejiang Hospital, Hangzhou, China; 2 Laboratory Medicine Center, Zhejiang Center for Clinical Laboratory, Zhejiang Provincial People's Hospital, People's Hospital of Hangzhou Medical College, Hangzhou, China; 3 Laboratory Medicine Center, Department of Clinical Laboratory, Zhejiang Provincial People's Hospital, Affiliated People's Hospital, Hangzhou Medical College, Hangzhou, China; 4 Department of Respiratory and Critical Care Medicine, Sir Run Run Shaw Hospital, Zhejiang University School of Medicine, Hangzhou, China; 5 Department of Biochemistry, HMU Health and Medical University Erfurt, Erfurt, Germany; 6 Department of Biochemistry and Molecular Biology, University of Southern Denmark, Odense, Denmark; 7 Department of Critical Care Medicine, Sir Run Run Shaw Hospital, Zhejiang University School of Medicine, Hangzhou, China; 8 Department of Clinical Laboratory, Affiliated Hangzhou First People's Hospital, School of Medicine, Westlake University, Hangzhou, China; 9 Department of Infectious Diseases, Sir Run Run Shaw Hospital, Zhejiang University School of Medicine, Hangzhou, China; 10 Zhejiang Provincial Engineering Research Center of Innovative Instruments for Precise Pathogen Detection, Hangzhou, China

**Keywords:** *Acinetobacter baumannii*, cefiderocol, genomic screening, promoter mutations, resistance mechanism

## Abstract

**Introduction:**

Carbapenem-resistant *Acinetobacter baumannii* (CRAB) poses a global health threat due to limited therapeutic options. Cefiderocol, a novel siderophore-conjugated cephalosporin, demonstrates potent *in vitro* activity against a variety of Gram-negative bacteria, including CRAB. However, cefiderocol's recent introduction into clinical practice means that while several resistance mechanisms have been identified, their full characterization remains incomplete, and novel mechanisms are likely to emerge as clinical use expands.

**Methods:**

In this study, *in vitro* evolution experiments were performed under cefiderocol pressure using a clinical CRAB isolate as the parental strain to investigate cefiderocol resistance mechanisms.

**Results:**

Genomic comparison and mutant reconstruction revealed that mutations in *pbp1b* (G271R) and the intergenic region upstream of *piuA* (-6_-8del) conferred a 4-fold increase in cefiderocol MIC. *In silico* predictions and EMSA confirmed that the intergenic mutation occurred within the *piuA* promoter region. qRT-PCR analysis indicated that the *piuA* promoter mutation significantly reduced *piuA* expression. This downregulation of *piuA* expression impaired cefiderocol transport, leading to reduced susceptibility to cefiderocol. A search across publicly deposited genome sequences identified ten *piuA* promoter mutations in *A. baumannii*. Among these, the−-6_-8del was the most commonly observed variant, which was primarily associated with ST2^Pas^/ST457^Oxf^, ST2^Pas^/ST208^Oxf^, and ST499^Pas^. Functional characterization of the−-6_-8del variant and three additional promoter mutations (-18del, A-17T&-18del, A-17T&-14del) confirmed suppression of *piuA* expression and reduction of cefiderocol susceptibility.

**Discussion:**

Combining *in vitro* evolution with publicly available *A. baumannii* genome sequences, this study demonstrates promoter-mediated *piuA* downregulation as a cefiderocol resistance mechanism in *A. baumannii*, providing a genomic target for predicting cefiderocol resistance.

## 1 Introduction

*Acinetobacter baumannii*, a formidable pathogen responsible for severe infections including bacteremia, pneumonia, meningitis, and urinary tract infections, has emerged as a critical global healthcare challenge. Its propensity for widespread antibiotic resistance severely limits therapeutic options (Peleg et al., 2008). Carbapenems, once considered last-resort agents against *A. baumannii*, are increasingly ineffective: data from China antimicrobial surveillance network (CHINET, http://www.chinets.com) revealed alarmingly high resistance rates in China for 2025, with 71.9% and 72% of isolates resistant to meropenem and imipenem, respectively. In response to the growing threat of untreatable infections, the World Health Organization (WHO) designated Carbapenem-resistant *Acinetobacter baumannii* (CRAB) as a “critical” priority pathogen in 2024, highlighting the urgent unmet medical need (Sati et al., 2025). While colistin and tigecycline remain options for CRAB infections, their clinical utility is hampered by significant toxicity concerns (nephrotoxicity, neurotoxicity) and the emergence of resistance (Bailey et al., 2022; Liu et al., 2024).

Cefiderocol, a novel siderophore-conjugated cephalosporin, represents a significant advancement in combating multidrug-resistant Gram-negative pathogens. Its unique “Trojan horse” mechanism exploits bacterial iron-uptake pathways: by binding ferric iron, it is actively transported across the outer membrane into the periplasmic space via TonB-dependent transporters (TBDTs) (Simner and Patel, 2020). This strategy achieves high periplasmic concentrations, endowing cefiderocol with potent *in vitro* activity against a broad spectrum of Gram-negative bacteria, including carbapenem-resistant isolates (Candel et al., 2022). Surveillance studies like SIDERO-CR demonstrated promising susceptibility rates, with 90.9% of CRAB isolates susceptible to cefiderocol (Yamano, 2019).

Nevertheless, the emergence of cefiderocol resistance in *A. baumannii* clinical isolates poses a significant threat. Known resistance mechanisms include the production of certain β-lactamases (e.g., PER-type, NDM-type) which can hydrolyze cefiderocol (Kohira et al., 2020; Poirel et al., 2021; Gaillot et al., 2023). Crucially, given its siderophore moiety, perturbations in iron acquisition systems play a pivotal role. Deficiencies in specific TBDTs responsible for siderophore uptake have been linked to resistance. For instance, loss-of-function mutations in *piuA* in *Pseudomonas aeruginosa* or in *cirA* and *fiu* in *Escherichia coli* resulted in significant increases (16-fold) in cefiderocol MICs (Ito et al., 2018). Clinical evidence corroborates this: a study in Taiwan found *piuA* deficiency in the majority (13 out of 21) of cefiderocol-resistant *A. baumannii* isolates (Yamano et al., 2022). Longitudinal study by Findlay et al. documented reduced *piuA* expression (down to 0.35-fold) correlating with a 32-fold MIC increase in CRAB isolates recovered from a patient after cefiderocol treatment (Findlay et al., 2024). While these findings underscore the importance of PiuA function and expression levels in cefiderocol susceptibility, the precise molecular mechanisms driving the downregulation of *piuA* expression in *A. baumannii*, particularly those involving mutations in regulatory regions, remain poorly understood.

To elucidate the mechanisms underlying cefiderocol resistance in CRAB, we employed *in vitro* evolution experiments. Through this approach, we identified a novel 3-bp deletion mutation specifically located within the promoter region of *piuA*, a gene encoding a key TBDT on the outer membrane. Concurrently, we screened natural *piuA* promoter variants from the NCBI database and validated their effects on cefiderocol susceptibility. Our *in vitro* evolution experiments elucidated the mechanisms of cefiderocol resistance in *A.baumannii*, which publicly deposited sequence databases confirm as clinically relevant (Figure S1). These insights provide an additional mechanism for predicting cefiderocol susceptibility from whole-genome sequences.

## 2 Materials and methods

### 2.1 Bacterial isolates, culture conditions and antimicrobial susceptibility testing

All bacterial isolates (Table S1) were cultured overnight at 37 °C on Mueller-Hinton agar (MHA) or in Mueller-Hinton broth (MHB; Oxoid, Hampshire, UK). Minimum inhibitory concentrations (MICs) were determined by broth microdilution in accordance with Clinical and Laboratory Standards Institute (CLSI) guidelines (CLSI, 2025). Cefiderocol susceptibility was assessed in iron-depleted cation-adjusted Mueller-Hinton broth (ID-CAMHB), prepared by adding the Chelex 100 Resin (Bio-Rad, USA; as specified in CLSI M100). Susceptibility to other antimicrobials was determined in standard cation-adjusted Mueller-Hinton broth (CAMHB). ATCC 25922 serves as the quality control strain.

### *2.2 In vitro* evolution experiment

Serial passage of strain XH386 under escalating cefiderocol pressure was performed in three independent biological replicates as described (Friedman et al., 2006). Briefly, single colonies of XH386 were separately inoculated into 2 mL of ID-CAMHB containing cefiderocol at 0.5 × MIC (0.25 μg/mL). Cultures were incubated at 37 °C for 24 h, then diluted 1:100 into fresh ID-CAMHB containing twice the previous cefiderocol concentration. This cycle was repeated daily until growth cessation. Cultures from sequential passages were designated XH386-D1 through XH386-D10 and cryopreserved at −80 °C. From each replicate lineage at passage D10, a single randomly selected colony was isolated and cryopreserved, designated XH386E1 (replicate 1), XH386E2 (replicate 2), and XH386E3 (replicate 3).

### 2.3 Genomic DNA sequencing and analysis

Genomic DNA was extracted using the QIAamp DNA Mini Kit (Qiagen, Valencia, CA, USA). Whole-genome sequencing was performed on the Illumina HiSeq™ 2000 platform (Illumina, San Diego, CA, USA). Sequence reads were mapped to the reference genome of parental strain XH386 (GenBank accession: CP010779.1) using breseq v0.33.0 for mutation identification (Deatherage and Barrick, 2014). All candidate mutations were validated by PCR amplification of target regions followed by Sanger sequencing.

A total of 8,647 *A. baumannii* genomes annotated by the NCBI RefSeq were retrieved from the NCBI Assembly database. The genome sequences were aligned against the reference *piuA* promoter sequence from strain XH386, which comprised 61 bp upstream of *piuA* and the first 39 bp of the *piuA* coding sequence, totaling 100 bp, using Snippy-multi. Genomes in which the *piuA* promoter region could not be unambiguously identified were excluded. A core genome alignment was conducted using a random set of 318 *A. baumannii* genomes harboring the wild-type *piuA* promoter and all 317 detected genomes carrying the mutant *piuA* promoter. Panaroo v1.3 (Tonkin-Hill et al., 2020) was run with a 95% core-genome threshold and strict cleaning mode, using MAFFT for multiple sequence alignment (Katoh et al., 2002), and only core gene alignments were retained for downstream analysis. Core-genome alignments were then used to construct maximum likelihood phylogenetic trees using IQ-TREE2 v2.3.5 (Minh et al., 2020) and visualized using iTOL v7 (Letunic and Bork, 2024). Genomic annotation was performed using Prokka v1.13 (Seemann, 2014). Multilocus sequence typing on the Center for genomic epidemiology website was used to scheme sequence type (ST).

### 2.4 Genetic manipulation

#### 2.4.1 Reconstruction experiments

Reconstruction of identified mutations was performed via allelic exchange as described (Amin et al., 2013; Xu et al., 2019). Purified PCR products containing target mutations (amplified using XH386E2 genomic DNA as template) were cloned into *Bam*HI/*Xba*I-digested pMo130-Hyg^R^ vector using the Hieff Clone^®^ Plus One Step Cloning Kit (Yeasen, China). Recombinant plasmids were electroporated into XH386, with transformants selected on hygromycin (300 μg/mL). Allelic replacement was induced by culturing in Luria-Bertani broth containing 20% sucrose to facilitate a second crossover event. Successful recombinant was confirmed by PCR and Sanger sequencing (primers in Table S2).

#### 2.4.2 *piuA* gene knockout

Upstream and downstream fragments flanking *piuA* were PCR-amplified and simultaneously ligated into *Bam*HI/*Xba*I-linearized pMo130-Hyg^R^ using the Hieff Clone^®^ Plus Multi One Step Cloning Kit (Yeasen, China). The resulting construct pMo130-Hyg^R^-Δ*piuA* was electroporated into XH386. Knockout mutants were selected via the hygromycin and sucrose counter-selection protocol described above.

#### 2.4.3 Site-directed mutagenesis

The native *piuA* promoter sequence was synthesized and cloned into pUC19. Site-directed mutagenesis was performed using the Hieff Mut™ Kit (Yeasen, China) to introduce specific substitutions. Mutant promoters were amplified by inverse PCR using mutation-containing primers (Table S2), then subcloned into pMo130-Hyg^R^. Promoter-variant strains were generated via the allelic exchange protocol.

### 2.5 Quantitative reverse transcription PCR (qRT-PCR)

Single colonies of parental strain XH386 and *piuA* promoter-variants were cultured in ID-CAMHB to mid-log phase. Total RNA was extracted using the PureLink™ RNA Mini Kit (Thermo Fisher Scientific, Waltham, MA, USA) with on-column DNase I treatment. RNA concentration was measured by Nanodrop. For each sample, 2 μg total RNA was reverse-transcribed using the PrimeScript™ RT Reagent Kit with gDNA Eraser (Takara Bio, Otsu, Japan).

qPCR amplification was performed in technical triplicates using TB Green™ Premix Ex Taq™ II (Takara Bio) on a LightCycler^®^ 480 II instrument (Roche Diagnostics, Mannheim, Germany). Reaction conditions: 95 °C/30 s; 40 cycles of 95 °C/5 s, 60 °C/60 s; followed by melt curve analysis. Three independent biological replicates were analyzed per strain. The *rpoB* gene served as the reference gene, and XH386 served as calibrator. Relative expression was calculated by the 2^−Δ*ΔCt*^ method. Primers are listed in Table S2.

### 2.6 Growth rate determination

Overnight cultures of each strain were diluted 1:1000 in MHB or iron-depleted MHB (MHB supplemented 250 μM 22′-dipyridyl [DIP]). Aliquots (200 μL) of diluted cultures were dispensed into triplicate wells of a 100-well honeycomb plate. Growth kinetics were monitored at 37 °C with continuous shaking using a Bioscreen C MBR system (Oy Growth Curves Ab Ltd., Finland), with optical density at 600 nm (OD_600_) recorded at 5-min intervals for 20 h. Three independent biological replicates were performed. Growth capacity was evaluated by calculating the area under the growth curve (AUC) using the GraphPad Prism. The relative growth reduction under iron-depleted conditions was defined as: growth reduction (%) = 100 × (AUC_MHB+DIP_ **–** AUC_MHB_) / AUC_MHB_.

### 2.7 Electrophoretic mobility shift assay (EMSA)

**RNA polymerase-promoter complex (RPo) formation experiment**. RNA polymerase (RNAP) was purified from BL21(DE3) cells (Thermo Fisher Scientific, USA) co-transformed with plasmids pGEMD and pIA900, following the protocol described by Shi et al. (2022). Binding reactions (20 μL) contained: buffer (40 mM Tris-HCl pH 8.0, 50 mM NaCl, 10 mM MgCl_2_, 5% glycerol), 40 nM *piuA* promoter DNA fragment, 0 or 80 nM RNAP. After 20-min incubation at 37 °C, 0.03 mg/mL heparin was added and incubated for 2 min at 22 °C to suppress non-specific binding. Then samples were resolved on 5% native polyacrylamide gels (29:1 acrylamide: bis-acrylamide) in 0.5 × TBE buffer (45 mM Tris-borate, pH 8.0, 1 mM EDTA) at 100 V for 90 min at 4 °C. Nucleic acids were stained with 4S Red Plus Nucleic Acid Stain (Sangon Biotech, China) for 20 min. Gel images were acquired using the ImageLab™ system (Bio-Rad Laboratories, USA) under red fluorescence mode.

**Fur binding experiments**. The putative Fur binding sites were PCR-amplified from XH386 or XH386 P*piuA*^−6_−8del^ genomic DNA and purified by using a DNA Clean & Concentrator kit (Zymo Research, Orange County, CA, USA). The reaction mixtures (20 μl) consisted of 150 mM NaCl, 20 mM Tris-HCl pH 8.0, 0.1 mg/mL bovine serum albumin, 10% glycerol, 200 μM Fe^2+^, 1 μM DNA probe and purified Fur protein (0, 10, 20, 40, 80 100 and 200 μM) were incubated at 37 °C for 30 min. Then, samples were separated on a 5% native polyacrylamide gel. Electrophoresis and detection were performed as described above.

### 2.8 Statistical analysis

All experiments were performed with at least three independent biological replicates, each containing three technical replicates. Normality of data distribution was assessed using the Shapiro-Wilk test, and homogeneity of variances was confirmed using Levene's test. Pairwise comparisons between two groups were performed using unpaired two-tailed Student's *t*-tests. A *P*-value < 0.05 was considered statistically significant. All statistical analyses were performed using GraphPad Prism.

## 3 Results

### 3.1 Identification of cefiderocol resistance-associated mutations

To elucidate cefiderocol resistance mechanisms, three parallel *in vitro* evolution experiments were performed using CRAB XH386 as the parental strain. After serial passage under escalating cefiderocol pressure, three high-level resistant clones (XH386E1, XH386E2, XH386E3; MIC >128 μg/mL) were isolated. The comparison of the whole genome sequences of the evolved cefiderocol-resistant strains with the parental XH386, revealed mutants with mutations in genes coding for efflux pumps (AdeJ^T473P^, AdeJ^E675K^), iron uptake systems (PirA^Q7*^, BfnH^A − 79C^, P*piuA*^−6_−8del^), and the penicillin-binding protein (PBP1b^G271R^). Interestingly, mutations occurred in both gene coding region and intergenic regions upstream of the protein coding sequences (Table 1).

**Table 1:** Mutation profile of evolved strains.

Strains	Position	Mutation	Annotation	Gene	Description
XH386E1	740875	ACC → CCC	T473P	TE32_03565←	AdeJ
	1247179	GGA → AGA	G271R	TE32_05710←	PBP1b
	2939679	CAG → TAG	Q7^*^	TE32_14205←	PirA
XH386E2	2110638	A → C	intergenic(-79/+7)	TE32_10010←/←TE32_10015	BfnH/BfnG
	3356589	(TGGTCGCAA)_2 → 1_	coding(527–535/1452 nt)	TE32_16225←	AbrP
	3536211	(ATT)4 → 3	intergenic(-380/-33)	TE32_17055←/ → TE32_17060	trigger factor/PiuA
XH386E3	740269	GAA → AAA	E675K	TE32_03565←	AdeJ
	1247179	GGA → AGA	G271R	TE32_05710←	PBP1b
	2938025	(ATCTT)_2 → 3_	coding(1673/2265 nt)	TE32_14205←	PirA

(i) Mutated nucleotides are highlighted in red. (ii) Arrows indicate the direction of gene transcription on the genome. ^*^Stop codon.

A functional validation of the above mutations was performed via allelic replacement and the antimicrobial susceptibility of recombinant strains. The data showed that mutations in *pbp1b* (G271R) and upstream of the *piuA* (-6_-8del) both independently increased cefiderocol MIC 4-fold from 0.5 μg/mL to 2 μg/mL. In contrast, mutations in *bfnH, adeJ, pirA, abrP* did not significantly alter cefiderocol susceptibility (Table 2).

**Table 2:** MICs of cefiderocol against induced-resistant strains and recombinant strains.

Strains	Cefiderocol MIC (μg/mL)
XH386	0.5
XH386E1	>128
XH386E2	>128
XH386E3	>128
XH386 AdeJ^T473P^	0.5
XH386 PBP1b^G271R^	2
XH386 PirA^Q7*^	0.5
XH386 BfnH^A − 79C^	0.5
XH386 AbrP^179_181del^	0.5
XH386 P*piuA*^−6_−8del^	2
XH386ΔPiuA	2
XH386 AdeJ^T473P^PBP1b^G271R^	2
XH386 AdeJ^T473P^PirA^Q7*^	0.5
XH386 PBP1b^G271R^PirA^Q7*^	2
XH386AdeJ^T473P^PBP1b^G271R^PirA^Q7*^	2
XH386 P*piuA*^179_181del^AbrP^179_181del^	2
XH386 P*piuA*^A − 27T^	0.5
XH386 P*piuA*^−21_−22insT^	0.5
XH386 P*piuA*^−18del^	2
XH386 P*piuA*^A − 17T, −18del^	2
XH386 P*piuA*^A − 17T, −14del^	2
XH386 P*piuA*^T − 16G^	0.5
XH386 P*piuA*^T − 15C^	1
XH386 P*piuA*^−5_−6insATT^	0.5
XH386 P*piuA*^T − 9A^	0.5

^*^Stop codon.

Double-site or multi-site mutations were then constructed in the genome of the parental strain XH386 with the goal to identify the mechanisms of the high-level cefiderocol resistance observed in the evolved resistant strains. However, despite creating mutations in all three sites (AdeJ^T473P^, PBP1b^G271R^ and PirA^Q7*^) or in two positions (P*piuA*^−6_−8del^ and AbrP^179_181del^), the MICs of the mutant strain did not exhibit the same level of resistance as the isolates obtained from the evolution experiments (Table 2), indicating additional yet unidentified mechanisms.

### 3.2 *piuA* promoter mutation downregulates expression and reduces cefiderocol susceptibility

**Figure 1:**
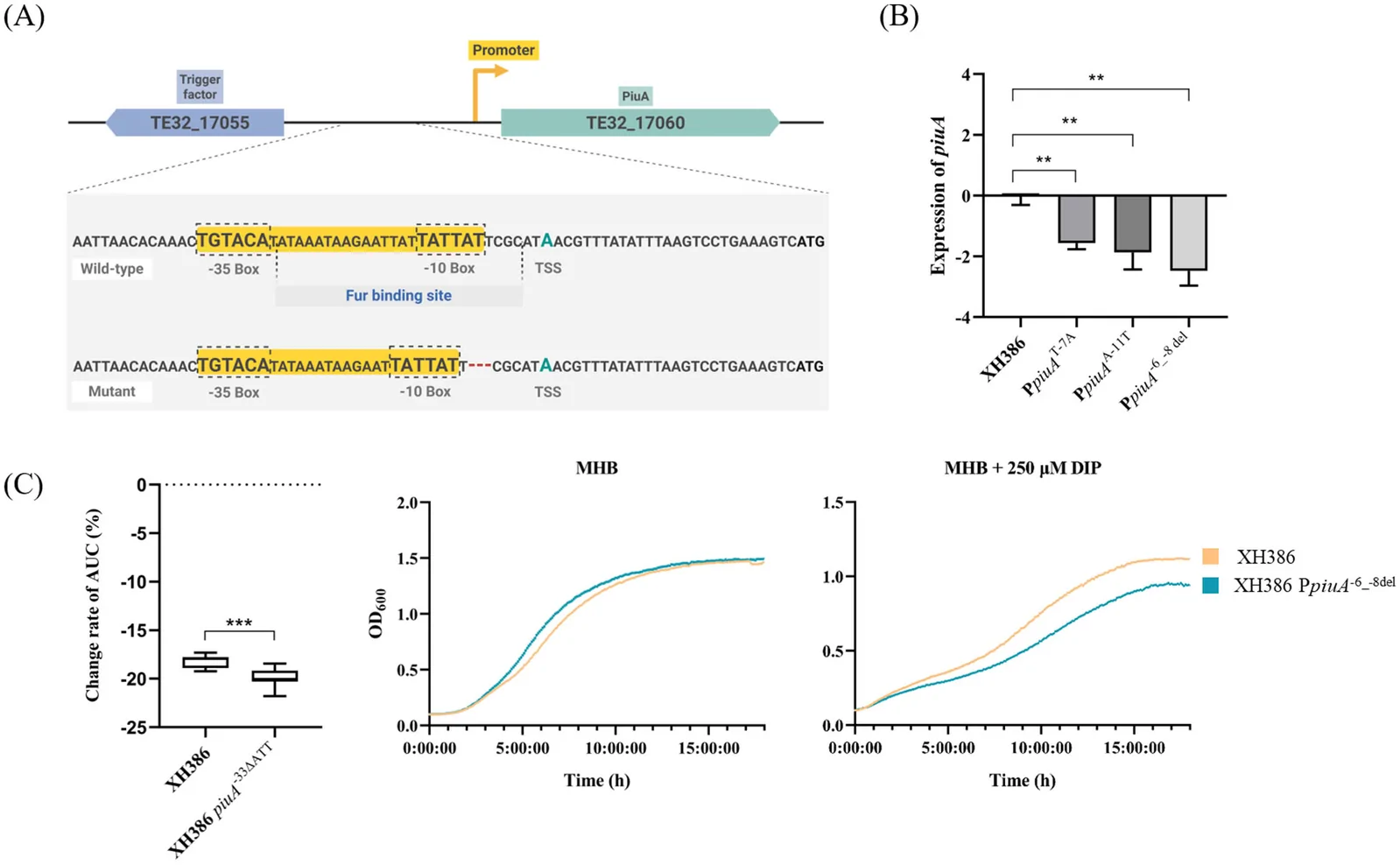
Mutations in the *piuA* promoter reduce *piuA* expression and the strain tolerance to iron-restricted conditions. **(A)** Schematic representation of the *piuA* promoter region. The predicted promoter region is located between −34 to −7 upstream of TSS (highlighted by orange rectangles). The predicted −35 Box and −10 Box are indicated by rectangles. The predicted Fur binding box is indicated by dotted lines. **(B)** *piuA* expression of XH386 P*piuA*^T − 7A^, XH386 P*piuA*^A − 11T^ and XH386 P*piuA*^−6_−8del^ compared to XH386. **P < 0.01 **(C)** Growth of wild-type XH386 and the mutant XH386 P*piuA*^−6_−8del^ under iron-replete (Middle panel) and iron-depleted (Right panel) conditions. The area under the curve (AUC) change rate (Left panel), calculated as 100% × (AUC_MHB+DIP_ - AUC_MHB_) / AUC_MHB_, quantifying the relative impact of iron depletion on bacterial growth and reflecting the strain's tolerance to iron-restricted conditions. ***P < 0.001.

In one of the evolved resistant strains, XH386E2, we identified a 3-bp deletion at positions −33 to −35 upstream of the *piuA* gene, reducing ATT tandem repeats from four to three units (Figure 1A). A subsequent *in silico* promoter analysis using BPROM and PromoterHunter (http://www.phisite.org/) identified putative transcription start site (TSS) of *piuA* and promoter (P*piuA*:5′-TGTACA-N16-TATTAT3′) spanning −34 to −7 bp relative to the TSS (Figure 1A), which positions the identified 3-bp deletion at 6 to 8 bp upstream of the TSS (named P*piuA*^−6_−8del^).

To functionally validate this region and confirm its role as a promoter, we performed RPo formation experiments, which confirmed that the polymerase binds to the wild-type *piuA* (P*piuA*) sequence (Figure S2B). Two variants were then created at conserved bases within the predicted −10 box: T-7A (“TATTAA”) and A-11T (“TATTAT”). Both promoter mutants exhibited significant downregulation of *piuA* transcription under iron-depleted conditions compared to the parental XH386 (*P* < 0.01, Figure 1B). functionally validating *piuA* promoter. The differences in *piuA* expression between XH386 and XH386 P*piuA*^−6_−8del^ under the condition of iron-limitation were assessed using qRT-PCR. Here, the expression of *piuA* in XH386 was significantly higher than that of XH386 P*piuA*^−6_−8del^ (Figure 1B). Interestingly, the *piuA* knockout strain and P*piuA*^−6_−8del^ mutant showed identical cefiderocol MICs (2 μg/mL, Table 2). These data demonstrate that promoter mutation (-6_-8 deletion) significantly reduces *piuA* expression, and conferring the strong reduction in cefiderocol susceptibility.

### 3.3 Impaired iron acquisition in the *piuA* promoter mutant

To assess functional consequences of the *piuA*^−6_−8del^ mutation, growth kinetics of the parental XH386 and mutant strains were compared in iron-replete (CAMHB) and iron-depleted media. The tolerance to iron deficiency environment is reflected by the change rate of area under curve (AUC) in two conditions. Quantitative analysis revealed that the *piuA*^−6_−8del^ mutant exhibited significantly reduced adaptation to iron-depleted conditions compared to parental XH386 (−18.20% ± 0.67% *vs*. −19.94% ± 0.95%, ΔAUC_ID − CAMHB/CAMHB_ unpaired *t*-test, *P* < 0.001), demonstrating impaired iron scavenging capacity (Figure 1C).

### 3.4 Clinically prevalent *piuA* promoter variants confer cefiderocol resistance via transcriptional repression

**Figure 2:**
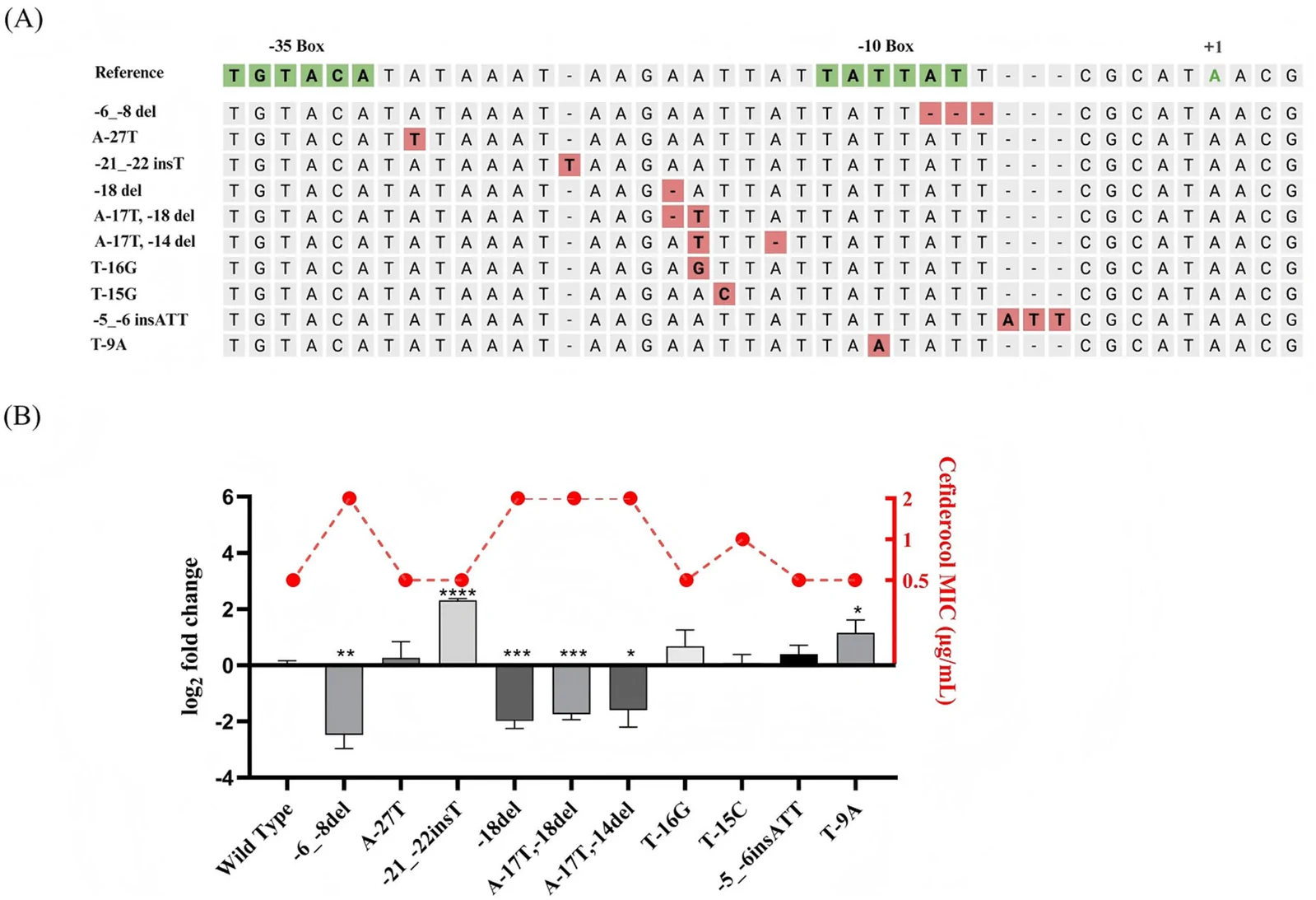
*piuA* promoter variants and their effects on gene expression. **(A)** Sequence alignment of all *piuA* promoter variants retrieved from the NCBI database. The conserved −10 and −35 boxes are highlighted. **(B)** Measurement of *piuA* expression levels driven by the different promoter variants. Promoter with site-directed mutations at positions−18del, A-17T&-18del and A-17T&-14del significantly reduced *piuA* expression compared to the wild-type promoter. *P < 0.05, **P < 0.01, ***P < 0.001, ****P < 0.0001.

To assess the clinical relevance of the mutations in the *piuA* promoter which we obtained in a lab-setting, we analyzed 8,647 *A. baumannii* genomes of clinical isolates deposited in the NCBI database, in order to address if any of those contain similar genetic changes. While the *piuA* promoter sequence was absent or could not be aligned in several deposited genomes, we observed ten distinct *piuA* promoter variants across the remaining 8,368 genomes (Figure 2A). The sequence identical to strain XH386 predominated and was present in 8,051 of the 8,368 sequences (96.2% prevalence), the variant we obtained in our evolution experiment (the−-6_-8 deletion) was found in 301 (3.60% prevalence). Nine additional minor variants collectively accounted for about 0.2% of clinical isolates.

We genetically reconstructed all the other nine promoter variants in the wild-type XH386 background and quantified their functional impact. Under iron-depleted conditions, three of the promoter variants P*piuA*^−18del^, P*piuA*^A − 17T&−14del^ and P*piuA*^A − 17T&−18del^ significantly suppressed *piuA* expression by 74.3 ± 2.9% (*P* = 0.0004), 70.0 ± 2.4% (*P* = 0.0003), and 65.3 ± 7.2% (*P* = 0.0109), respectively (Figure 2B). This transcriptional defect translated into substantially elevated cefiderocol MICs (Table 2) of XH386 P*piuA*^−18del^, XH386 P*piuA*^A − 17T&−18del^, and XH386 P*piuA*^A − 17T&−14del^ from 0.5 μg/mL to 2 μg/mL (4-fold increase *vs*. parental XH386).

### 3.5 Phylogenetic distribution of *piuA* promoter mutations

**Figure 3:**
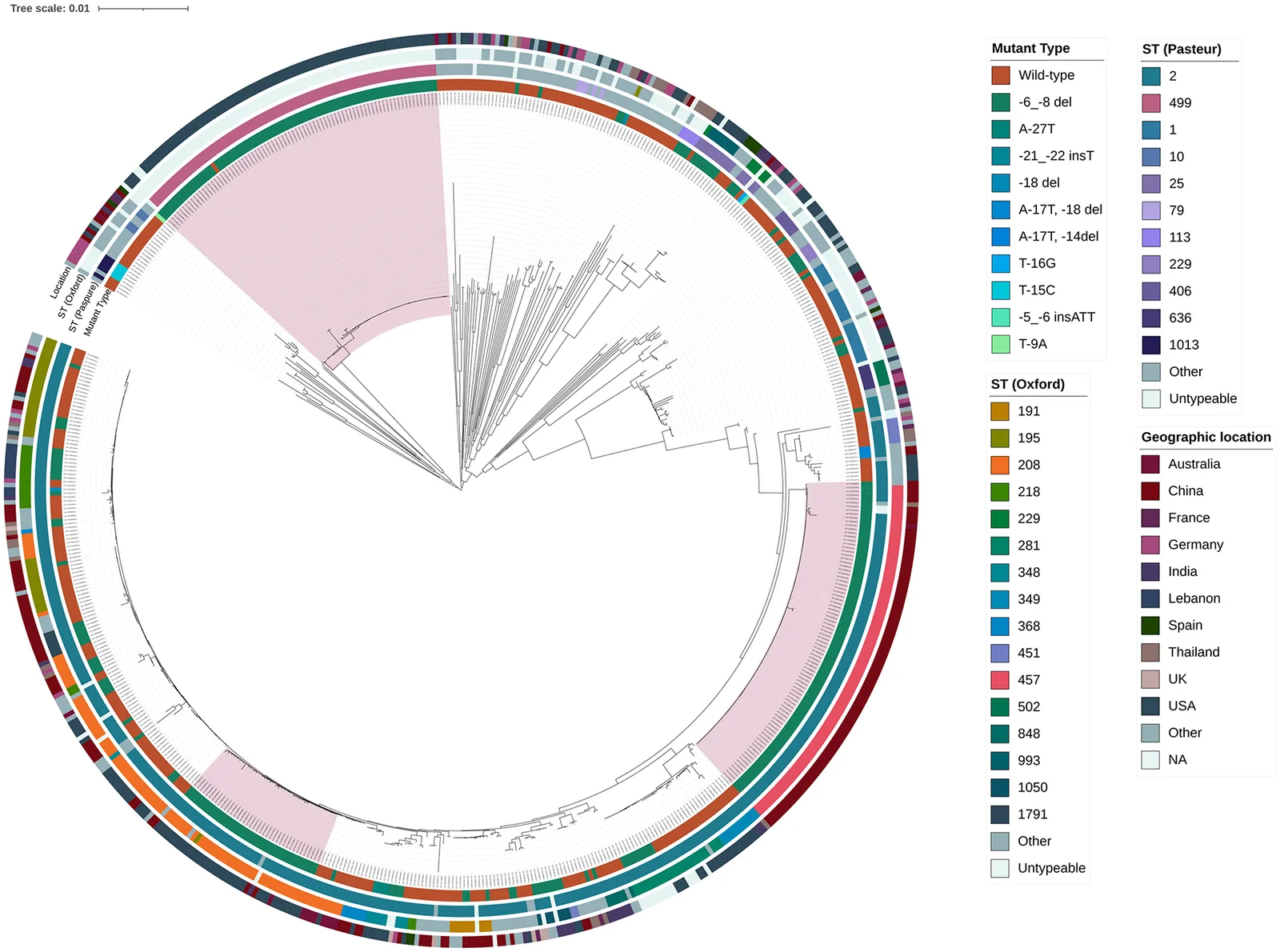
Phylogenetic analysis of *A. baumannii* isolates carrying *piuA* promoter mutations. Maximum likelihood phylogenetic tree constructed from core-genome alignments of 635 *A. baumannii* genomes, comprising 317 strains with mutant *piuA* promoters and 318 randomly selected wild-type strains. The tree was rooted at the midpoint. Annotated layers from inner to outer rings indicate: (i) *piuA* promoter mutation type (colored according to the legend). (ii) Pasteur sequence type. (iii) Oxford sequence type, and (iv) geographic region of isolation. Three major clades enriched for the P*piuA*^−6_−8del^ mutation are highlighted (Clades I-III). Clade I predominantly comprises ST499^Pas^ strains from the United States; Clade II consists primarily of ST2^Pas^/ST457^Oxf^ strains from China; Clade III is mainly composed of ST2^Pas^/ST208^Oxf^ strains from the United States, with additional isolates from China, India, and Australia.

To define the genomic epidemiology of the identified *piuA* promoter mutation, we constructed a phylogenetic tree from a comprehensive dataset comprising 317 *piuA* promoter mutants and 318 randomly selected wild-type *A. baumannii* genomes (Figure 3). The dataset contains strains with 90 distinct Pasteur sequence types (STs), with ST2 ^Pas^ being the most prevalent (370/635), followed by ST499 ^Pas^ (79/635). The ST2^Pas^ background encompassed both wild-type strains and various promoter mutant types (-6_-8del, A-27T,−-18del, A-17T&-18del, A-17T&-14del).

The predominant P*piuA*^−6_−8del^ mutation (n=301) was associated with specific genetic backgrounds. Under the Pasteur scheme, P*piuA*^−6_−8del^ mutants were predominantly associated with ST2 (185/301, 61.4%), ST499 (78/301, 25.9%), and ST25 (11/301, 3.7%). Corresponding analysis using the Oxford scheme identified ST457 (87/301, 28.9%) and ST208 (46/301, 15.3%) as the major types, with 97 strains that could not be assigned any sequence type.

Notably, phylogenetic reconstruction revealed that the P*piuA*^−6_−8del^ mutants clustered into three major clades: Clade I consisted predominantly of ST499^Pas^ (Oxford untypeable) strains, almost exclusively isolated from the United States. Clade II was characterized by ST2^Pas^/ST457^Oxf^ strains and was primarily comprised of isolates from China. Clade III, mainly comprising ST2^Pas^/ST208^Oxf^ strains, was largely found in the United States, with additional isolates from China, India, and Australia. Besides these distinct clusters, a number of mutants were interspersed throughout the phylogenetic tree alongside wild-type strains, without forming separate monophyletic groups.

## 4 Discussion

Cefiderocol has demonstrated potent *in vitro* activity against a broad spectrum of Gram-negative bacteria. Nevertheless, studies consistently report *A. baumannii* exhibits higher resistance rates and MIC_50/90_ values among clinically relevant pathogens. Five consecutive annual multinational (2014–2019) SIDERO-WT surveillance studies data indicated that *A. baumannii* exhibited the lowest susceptibility rate to cefiderocol at 96%, which was lower than those of *Enterobacterales* (99.8%), *Pseudomonas aeruginosa* (99.9%), and *S. maltophilia* (98.6%, Karlowsky et al., 2022). A multicenter study in China also revealed that *A. baumannii* exhibited the lower susceptibility rate to cefiderocol, which was lower than those of *P. aeruginosa* and *S. maltophilia*, but comparable to that of *Enterobacterales* (Kohira et al., 2023). Correspondingly, phase 3 trial CREDIBLE-CR indicated that numerically more deaths occurred in the cefiderocol group in the patient subset with *Acinetobacter* spp infections (Bassetti et al., 2021). Therefore, elucidating the mechanisms underlying *A. baumannii* resistance to cefiderocol is crucial for optimizing its clinical deployment and delaying the emergence of resistance.

To investigate potential cefiderocol resistance mechanisms, *in vitro* experimental evolution was performed under cefiderocol pressure. Mutations in *adeJ, pbp1b, pirA*, and others were identified in the evolved resistant strains and subsequently validated. Previous research has established that the construction of a *pbp3* gene mutation in *E. coli*, involving the insertion of YRIN or YRIK after proline at position 333, resulted in a 2-fold increase in the cefiderocol MIC (Sato et al., 2020). PBP serves as the target site for cefiderocol. Affinity binding assays demonstrate that cefiderocol binds to PBP1, PBP2, and PBP3 of *A. baumannii*, with IC_50_ values of 1.05, 2.31, and 0.67 μg/mL, respectively, indicating the strongest affinity for PBP3 (Ito et al., 2018). Although PBP3 exhibits the highest affinity for cefiderocol, the observed G271R substitution in PBP1b resulted in a 4-fold increase in MIC in our recombinant mutant, suggesting that alterations in PBP1b can also impact cefiderocol susceptibility. The G271 residue is located within the transglycosylase domain, and substitution of this glycine with arginine may affect the structural conformation or catalytic activity of PBP1b, potentially reducing its affinity for cefiderocol. However, detailed structural studies would be required to fully elucidate the molecular basis of this effect.

A frameshift mutation was observed in an evolved resistant strain which led to premature termination of the PirA protein. A previous report describes that *A. baumannii* strains with cefiderocol MIC ≥8 μg/mL exhibit reduced expression of *piuA* and *pirA* (Asrat et al., 2023); also, Saquib Malik et al. described PirA mutations which resulted in low expression of the protein in cefiderocol-resistant *A. baumannii* clinical isolates (Malik et al., 2020). Despite these observations, the mutant that we observed which results in the loss of PirA function did not significantly alter cefiderocol susceptibility. Another mutation was identified in *abrP*; this genetic change did not affect cefiderocol MIC but interestingly increased the MICs of ceftazidime and cefepime by ≥8 fold, indicating that cefiderocol selective pressure can co-select for resistance to other cephalosporins possibly due to their structural similarity.

**Figure 4:**
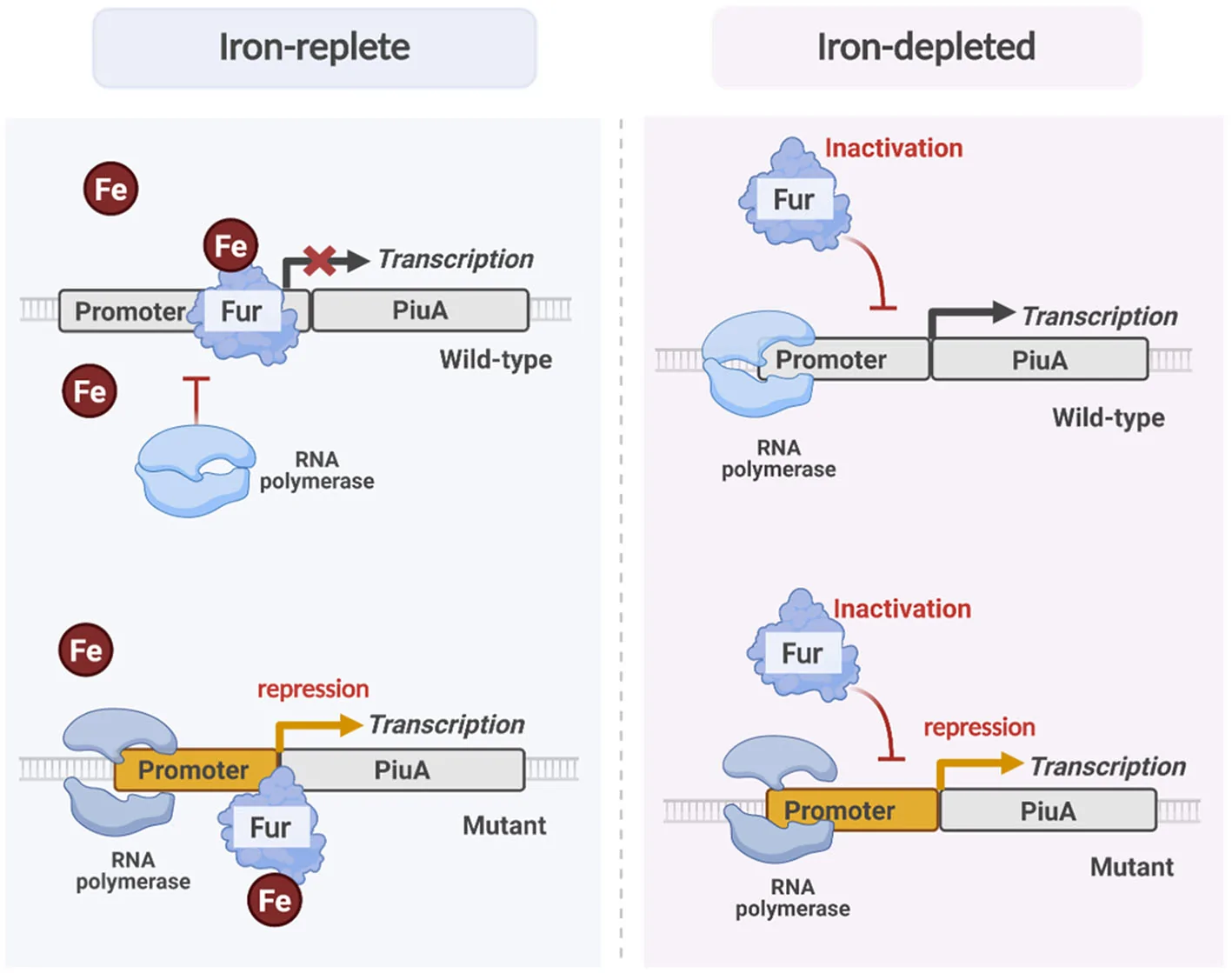
Working model for *piuA* regulation and the consequence of promoter mutation. Under iron-replete conditions, iron-bound Fur (Fe-Fur) is activated and binds to the Fur box upstream of the *piuA* gene, sterically hindering RNA polymerase (RNAP) recruitment and repressing *piuA* transcription. Under iron-depleted conditions, apo-Fur (iron-free) dissociates from the DNA, allowing RNAP access and activating *piuA*. In the mutant promoter the−-6_-8del mutation disrupts the Fur box and attenuates promoter activity. As a result, *piuA* expression is reduced regardless of iron availability, leading to reduced PiuA-mediated cefiderocol uptake and decreased susceptibility. Created with BioRender.com.

Aside from the mutations mentioned above, the focus of our study were mutations within the predicted promoter region of *piuA*, which had a clear effect on cefiderocol MIC. Although the MIC of 2 μg/mL remains below the CLSI susceptible breakpoint ( ≤ 4 μg/mL), it approaches the upper range of the cefiderocol MIC_90_ (1–2 μg/mL) reported for *A. baumannii* in global surveillance (Karlowsky et al., 2022), indicating that *piuA* promoter mutations may facilitate a shift toward reduced susceptibility that warrants clinical attention. Using the genetic backbone of the parental strain, we constructed the two most strongly conserved bases of the−10 Box,−11A and−7T (negative numbering denotes the nucleotide position upstream of the TSS at +1) to verify the function of the *piuA* promoter (Mueller et al., 2023). We observed that the mutations significantly reduced *piuA* expression, confirming the functional importance of this promoter region. Notably, iron limitation induced high PiuA levels in the parental strain but not in promoter mutant strains, with no significant induction observed (Figure S2A). This demonstrates that iron concentration is a key regulator of *piuA* expression. Bioinformatics analysis by Bart A (Eijkelkamp et al., 2011) predicted a putative Fur (Ferric Uptake Regulator) binding site (ATAAATAAGAATTATTATTATTCGC), which is overlapping the core promoter region of *piuA* in this study (Figure 1A). Fur is a transcriptional repressor that maintains intracellular iron homeostasis by repressing iron acquisition genes under iron-replete conditions. Our electrophoretical mobility shift assay (EMSA) results experimentally confirmed Fur binding to this specific sequence within the *piuA* promoter (Figure S2C). Furthermore, the 3-bp intergenic deletion identified in our evolved strains also altered the Fur box sequence. This study suggests that these mutataions impair cefiderocol resistance by dual mechanisms: (1) reducing basal promoter activity, and (2) disrupting Fur-mediated repression under iron-replete conditions. Consequently, the mutant strain fails to adequately upregulate PiuA expression under the iron-depleted conditions necessary for optimal cefiderocol uptake, leading to decreased susceptibility (Figure 4). This highlights the critical importance of iron availability at the site of action for cefiderocol efficacy. Meanwhile, growth kinetics analysis revealed significantly reduced adaptation to iron limitation in the promoter mutant, the concurrent deficiency in iron utilization and antibiotic influx establishes PiuA as a dual-function gatekeeper governing both bacterial iron homeostasis and susceptibility to siderophore-conjugated antibiotics.

As our study was performed employing a wet-lab evolution approach, we also addressed the question if our findings are of clinical relevance. To this end, we performed a large-scale genomic mining of NCBI sequence datasets which revealed distinct *piuA* promoter variants with differential regulatory activities. The most common non-wild-type variant observed was indeed our observed−-6_-8 deletion. Strains carrying this mutation were primarily isolated from the United States and China, but have also been widely identified in 16 other countries including Thailand, Lebanon, Germany, and Australia. In addition to its presence in ST2 and ST499 strains, the−-6_-8 variant was detected in 15 other distinct sequence types, such as ST25, ST113, and ST229. Phylogenetically, these mutants are distributed across three major clades, with isolation dates spanning from 2012 to 2023. Although we cannot entirely exclude the possibility of clonal dissemination within certain clades, the presence of this mutation across diverse geographic locations, distinct ST backgrounds, and extended time periods strongly suggests that the−-6_-8del mutation has arisen independently on multiple occasions in different genetic contexts, rather than resulting from a single clonal expansion event. The recurrent appearance of these promoter mutations across diverse lineages and geographic regions underscores their potential clinical relevance and reinforces the need for routine surveillance of the *piuA* promoter region in *A. baumannii* populations.

In addition to the 3-bp deletion, three other variants (-18del, A-17T&-18del, and A-17T &−14del) also significantly reduced *piuA* expression and decreased bacterial susceptibility. We propose that these effects are largely due to altered spacing between the−10 and−35 boxes: all four of the above-mentioned mutations shorten the spacer to 13 bp or 15 bp. Since the optimal spacer length for bacterial promoters is 16–18 bp (with 17 bp being ideal), and deviation from this optimal length has been shown to significantly impair promoter strength by affecting RNA polymerase binding and open complex formation (Hawley and McClure, 1983). Conversely, in variant XH386 P*piuA*^−21_−22insT^, the insertion restores the optimal 17-bp spacing, resulting in significant upregulation of *piuA* expression. Critically, these findings indicate that surveillance limited to PiuA coding mutations is insufficient; we strongly advocate for routine monitoring of promoter architecture in clinical isolates. This work fundamentally expands the scope of resistance surveillance targets for cefiderocol, establishing *piuA* regulatory elements as essential diagnostic markers.

This study has several limitations. RNA polymerase was shown to bind both the wild-type and mutant *piuA* promoter regions, but quantitative binding affinities were not determined, which limits inferences about affinity differences. Cefiderocol uptake was assessed indirectly through iron utilization rather than directly measured. Furthermore, although multiple mutations were identified and validated in the evolved strains, combinatorial reconstruction of these mutations did not fully recapitulate the high-level resistance phenotype, suggesting that additional, as-yet-unidentified mechanisms contribute to the high-level resistance.

Despite these limitations, the findings establish a mechanistic link between *piuA* promoter mutations and reduced cefiderocol susceptibility, providing a foundation for further investigations. Future work should systematically evaluate the prevalence and clinical impact of these promoter mutations among cefiderocol-resistant *A. baumannii* isolates and develop rapid diagnostic tools targeting this region for early resistance detection. Such quantitative biophysical and clinical studies, built upon the observations presented here, will be essential to fully elucidate the resistance mechanism and translate these findings into clinical practice.

## 5 Conclusion

In conclusion, under cefiderocol selective pressure, a deletion mutation (-6_-8del) occurred in the promoter region of the one of the TBDTs coding gene *piuA* in CRAB strain XH386. Under iron- depleted conditions, this 3-bp deletion reduces *piuA* expression, impairing both bacterial iron acquisition and cefiderocol transport, ultimately leading to decreased susceptibility to cefiderocol. Notably, this mutation was identified not only *in vitro*-induced resistant strains but also among 3.60% *A. baumannii* strains from global databases. Additionally, mutations (-18del, A-17T&-18del, and A-17T&-14del) within the promoter were also found to reduce *piuA* expression and confer decreased cefiderocol susceptibility. This study confirms that PiuA serves as a key transporter for cefiderocol and demonstrates that promoter mutations downregu-lating *piuA* expression represent a novel mechanism of resistance. These findings provide new genetic markers for surveillance of cefiderocol resistance in *A. baumannii*.

## Data Availability

The data presented in the study are deposited in the Sequence Read Archive repository, accession number PRJNA1375811.
