# Supplementary material for: PiuA promoter mutations confer cefiderocol resistance in Acinetobacter baumannii: from in vitro evolution to global genomic screening
Source: Front Microbiol. 2026 Sep 10;17:1899246. doi: 10.3389/fmicb.2026.1899246 (PMC13601296; doi:10.3389/fmicb.2026.1899246)
Supplement: Supplementary file 1 [file Image_1.pdf]

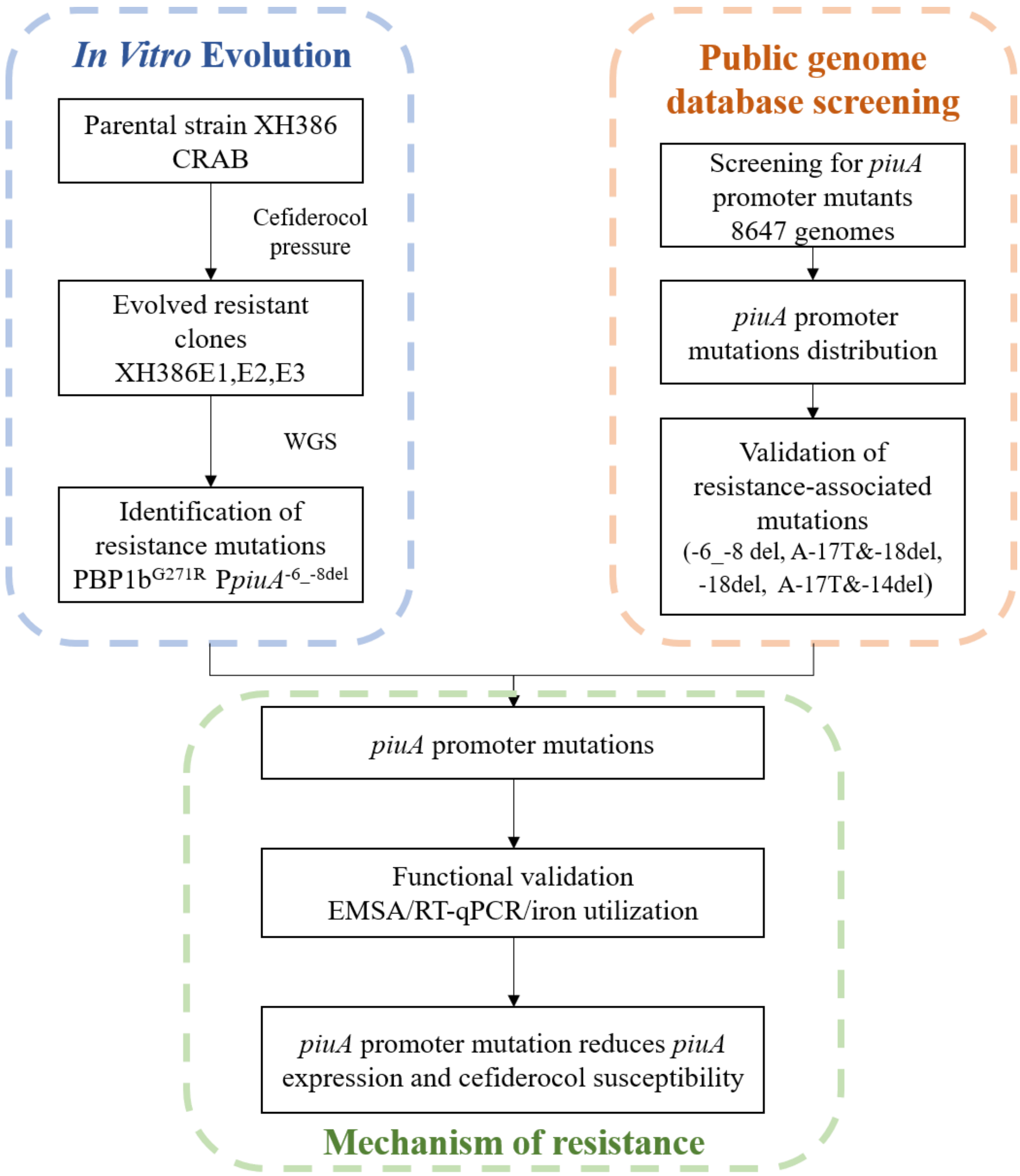

Figure S1. Overview of the study workflow for identifying and validating the cefiderocol resistance mechanism in *A. baumannii*.
