# Supplementary material for: PiuA promoter mutations confer cefiderocol resistance in Acinetobacter baumannii: from in vitro evolution to global genomic screening
Source: Front Microbiol. 2026 Sep 10;17:1899246. doi: 10.3389/fmicb.2026.1899246 (PMC13601296; doi:10.3389/fmicb.2026.1899246)
Supplement: Supplementary file 2 [file Image_2.pdf]

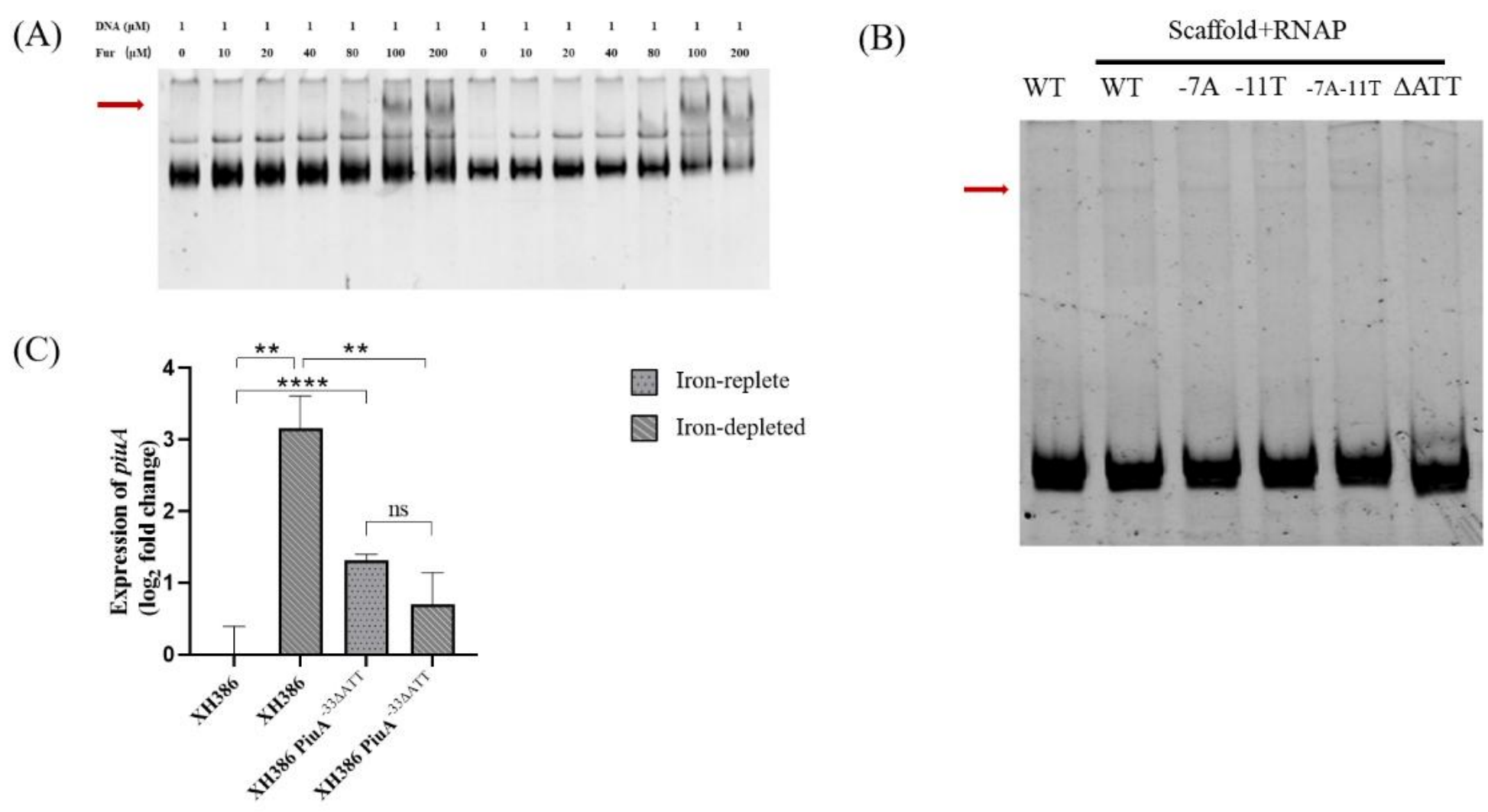

Figure S2. Iron-dependent regulatory mechanism of *piuA* expression. (A) EMSA demonstrates that the Fur protein binds to the *piuA* promoter region, with a stronger affinity for the wild-type sequence (Lane 1-7) compared to the mutant promoter (Lane 8-14). (B) RPo formation experiments demonstrated the binding of RNA polymerase to the predicted *piuA* promoter sequence. (C) Comparison of *piuA* expression between the wild-type XH386 and the XH386 P*piuA*$^{-6\_-8\ \mathrm{del}}$ cultured under iron-depleted and iron-replete conditions.
