# Supplementary material for: PiuA promoter mutations confer cefiderocol resistance in Acinetobacter baumannii: from in vitro evolution to global genomic screening
Source: Front Microbiol. 2026 Sep 10;17:1899246. doi: 10.3389/fmicb.2026.1899246 (PMC13601296; doi:10.3389/fmicb.2026.1899246)
Supplement: Supplementary file 3 [file Table_1.pdf]

Table S1. Description of plasmids and bacterial strains used in this study

Plasmid/Strain	Discription	Source
Plasmid		
pMo130-Hyg^{R}	Suicide plasmid with hygromycin resistance cassette	Lab stock
pMo130-*piuA*(up/down)	pMo130-Hyg^{R} carrying the upstream and downstream DNA fragments of the *piuA* gene, used for targeted knockout of the *piuA* gene.	This Study
pMo130-gene^{M}	pMo130-Hyg^{R} carrying mutation sites in AdeJ, AbrP, PBP1b, PirA, BfnH, or PiuA promoter, used for *in situ* construction of point mutations.	This Study
Strains		
XH386	Clinical isolation of *A. baumannii* strain	Lab stock
XH386E1, XH386E2, XH386E3	cefiderocol resistant strain mutation abtained from *in vitro* evolution experiment of XH386	This Study
XH386 AdeJ^{T473P}	AdeJ (T473P) mutation recombinant strain based on the genetic background of XH386	This Study
XH386 PBP1b^{G271R}	PBP1b (G271R) mutation recombinant strain based on the genetic background of XH386	This Study
XH386 PirA^{Q7*}	PirA (Q7*) mutation recombinant strain based on the genetic background of XH386	This Study
XH386 P*piuA*$^{-6\_-8del}$	PiuA promoter (-6 del ATT) mutation recombinant strain based on the genetic background of XH386	This Study
XH386 AbrP^{179_181del}	AbrP (179 del IAT) mutation recombinant strain based on the genetic background of XH386	This Study
XH386 BfnH^{A-79C}	BfnH (A-79C) mutation recombinant strain based on the genetic background of XH386	This Study
XH386 AdeJ^{T473P}PBP1b^{G271R}	XH386 with mutations in AdeJ (T473P) and PBP1b (G271R)	This Study
XH386 AdeJ^{T473P}PirA^{Q7*}	XH386 with mutations in AdeJ (T473P) and PirA(Q7*)	This Study
XH386 PBP1b^{G271R}PirA^{Q7*}	XH386 with mutations in PBP1b (G271R) and PirA(Q7*)	This Study
XH386 AdeJ^{T473P}PBP1b^{G271R}PirA^{Q7*}	XH386 with mutations in AdeJ (T473P), PBP1b (G271R) and PirA(Q7*)	This Study
XH386 P*piuA*$^{-6\_-8del}$ AbrP^{179_181del}	XH386 with mutations in PiuA promoter (-15 del ATT) and AbrP (179 del IAT)	This Study
XH386ΔPiuA	PiuA knockout strain of XH386	This Study
XH386 P*piuA*$^{A-27T,}$	PiuA promoter (A-27T) mutation recombinant strain based on the genetic background of XH386	This Study

Plasmid/Strain	Discription	Source
XH386 P$piuA^{-21_-22insT}$	PiuA promoter (-21_-22 ins T) mutation recombinant strain based on the genetic background of XH386	This Study
XH386 P$piuA^{-18del}$	PiuA promoter (-18 del) mutation recombinant strain based on the genetic background of XH386	This Study
XH386 P$piuA^{A-17T\&-18del}$	PiuA promoter (A-17T and -18 del) mutations recombinant strain based on the genetic background of XH386	This Study
XH386 P$piuA^{A-17T\&-14del}$	PiuA promoter (A-17T and -14 del) mutations recombinant strain based on the genetic background of XH386	This Study
XH386 P$piuA^{T-16G}$	PiuA promoter (T-16G) mutation recombinant strain based on the genetic background of XH386	This Study
XH386 P$piuA^{T-15C}$	PiuA promoter (A-15C) mutation recombinant strain based on the genetic background of XH386	This Study
XH386 P$piuA^{-5_-6insATT}$	PiuA promoter (-5_-6 ins ATT) mutation recombinant strain based on the genetic background of XH386	This Study
XH386 P$piuA^{T-9A}$	PiuA promoter (T-9A) mutation recombinant strain based on the genetic background of XH386	This Study
ATCC25922	*E.coli* model strain	Lab stock

Table S2 Primers used in this study

Primer Name	Sequence (5'→3')
Reconstruction experiments	
PiuA-P-F	CTGACCTGACCTGAGcggccgccctgcagcggatccTGCTTACCAATACGCCAAGC
PiuA-P-R	tgccgggagcagacaagcccgtcgcatgcatctagaATGCCAAACAAGACCTTTGCC
Inveri-PiuA-P-F	ATTTGAGCGCGTTGTTGAGC
Inveri-PiuA-P-R	TGCCAGGAACAGTACGCAAT
AdeJ-F	tgaccgtgctgacctgacctgagcggccgccctgcagATACCCGCGTTTTGACCAACA
AdeJ-R	gagcagacaagcccgtcgcatgcatctagagggatccACACAGCCGAAGCAGTTGAAC
Inveri-AdeJ-F	TTGGACGCACACCTACAAGA
Inveri-AdeJ-R	GTTACGGCCACTTCTCGCTC
PBP-F	tgaccgtgctgacctgacctgagcggccgccctgcagTTAAAAGTTGCACCCGCCAAG
PBP--R	gcagacaagcccgtcgcatgcatctagagggatccAACATTTGGTGCGACTGCTC
Inveri-PBP-F	CCATCTGGTTTTCACTTTCACGA
Inveri-PBP-R	ACTGGAGTTGCCCGTTTAGAA
PirA-F	tgaccgtgctgacctgacctgagcggccgccctgcagAGCTTTTGCTACCCCACCTTTT
PirA-R	agcagacaagcccgtcgcatgcatctagagggatccACAAAAATCAGCTTCCACGGC
Inveri-PirA-F	GCAGCCATTGCCATTTGTACT
Inveri-PirA-R	GGACACGGCGGTAACTGTAG
AbrP-F	ctgacctgacctgagcggccgccctgcagcggatcc CCCATGCACATTCAGTTGGC
AbrP-R	atgccgggagcagacaagcccgtcgcatgcatctaga CTAGTCGATGCTACAGCGGG
Inveri-AbrP-F	GAGACTAAAGCCGTGGCATC
Inveri-AbrP-R	ATAGTTGGCTTGTTGCCGGT
BfnH-P-F	ctgacctgacctgagcggccgccctgcagcggatccAACCTTTACGTTGGCCTGTT
BfnH-P-R	atgccgggagcagacaagcccgtcgcatgcatctagaGGAAAACCCGAAACCCACTT
Inveri-BfnH-P-F	CGTCAGGGAAACTAAAGCCCT
Inveri-BfnH-P-R	ACTCGCAATGAAGGGGAAAGT
Gene knockout	
PiuA-UP-F	ctgacctgacctgagcggccgccctgcagcggatccTCGGCAAATCCAATTAACACAA
PiuA-UP-R	ttgagttggaccattgagcgaagtacaccaTGTCGGTAAGGTCGTGTTCTAATT

Primer Name	Sequence (5’→3’)
PiuA-DW-F	acaattaaattagaacacgaccttaccgacaTGGTGTACTTCGCTCAATGGTC
PiuA-DW-R	gccgggagcagacaagcccgtcgcatgcatctagaCTGCTAGAACTGCACTACGGCC
Veri-PiuA-F	TTGCGGTCGAGCAAGTAGAG
Veri-PiuA-R	GTCTGGGCGTGCTTGTAAAC
Inveri-PiuA-F	TTGCGGTCGAGCAAGTAGAG
Inveri-PiuA-R	AGCTGAGTTCAAACGATGCC
Site-directed mutagenesis	
$PiuA^{T\text{-}7A}$-F	AAGAATTATTATTAATCGCATAACGTTTATATTTAAGTCCTGAAAGTC
$PiuA^{T\text{-}7A}$-R	TATGCGATTAATAATAATTCTTATTTATATGTACAGTTTGTGTTAATT
$PiuA^{A\text{-}11T}$-F	AAGAATTATTTTTATTCGCATAACGTTTATATTTAAGTCCTGAAAGTC
$PiuA^{A\text{-}11T}$-R	TATGCGAATAAAAATAATTCTTATTTATATGTACAGTTTGTGTTAATT
$PiuA^{T\text{-}7A\ A\text{-}11T}$-F	AAGAATTATTTTTAATCGCATAACGTTTATATTTAAGTCCTGAAAGTC
$PiuA^{T\text{-}7A\ A\text{-}11T}$-R	TATGCGATTAAAAATAATTCTTATTTATATGTACAGTTTGTGTTAATT
$PiuA^{-6\_-8del}$-F	AAGAATTATTATTCGCATAACGTTTATATTTAAGTCCTGAAAGTC
$PiuA^{-6\_-8del}$-R	TATGCGAATAATAATTCTTATTTATATGTACAGTTTGTGTTAATT
$PiuA^{A\text{-}27T}$-F	AAGAATTATTATTATTCGCATAACGTTTATATTTAAGTCCTGAAAGTC
$PiuA^{A\text{-}27T}$-R	TATGCGAATAATAATAATTCTTATTTAAATGTACAGTTTGTGTTAATT
$PiuA^{-21\_-22insT}$-F	TTAAGAATTATTATTATTCGCATAACGTTTATATTTAAGTCCTGAAAG
$PiuA^{-21\_-22insT}$-R	TATGCGAATAATAATAATTCTTAATTTATATGTACAGTTTGTGTTAAT
$PiuA^{-18del}$-F	AAGATTATTATTATTCGCATAACGTTTATATTTAAGTCCTGAAAGTC
$PiuA^{-18del}$-R	TATGCGAATAATAATAATCTTATTTATATGTACAGTTTGTGTTAATT
$PiuA^{A\text{-}17T,\ -18del}$-F	AAGTTTATTATTATTCGCATAACGTTTATATTTAAGTCCTGAAAGTC
$PiuA^{A\text{-}17T,\ -18del}$-R	TATGCGAATAATAATAAACTTATTTATATGTACAGTTTGTGTTAATT
$PiuA^{A\text{-}17T,\ -14del}$-F	AAGATTTTTATTATTCGCATAACGTTTATATTTAAGTCCTGAAAGTC
$PiuA^{A\text{-}17T,\ -14del}$-R	TATGCGAATAATAAAAATCTTATTTATATGTACAGTTTGTGTTAATT
$PiuA^{T\text{-}16G}$-F	AAGAAGTATTATTATTCGCATAACGTTTATATTTAAGTCCTGAAAGTC
$PiuA^{T\text{-}16G}$-R	TATGCGAATAATAATACTTCTTATTTATATGTACAGTTTGTGTTAATT
$PiuA^{T\text{-}15C}$-F	AAGAATCATTATTATTCGCATAACGTTTATATTTAAGTCCTGAAAGTC
$PiuA^{T\text{-}15C}$-R	TATGCGAATAATAATGATTCTTATTTATATGTACAGTTTGTGTTAATT

Primer Name	Sequence (5’→3’)
PiuA$^{-5_-6insATT}$-F	AGAATTATTATTATTATTCGCATAACGTTTATATTTAAGTCCTGAAAG
PiuA$^{-5_-6insATT}$-R	TGCGAATAATAATAATAATTCTTATTTATATGTACAGTTTGTGTTAAT
PiuA^{T-9A}-F	AAGAATTATTATAATTCGCATAACGTTTATATTTAAGTCCTGAAAGTC
PiuA^{T-9A}-R	TATGCGAATTATAATAATTCTTATTTATATGTACAGTTTGTGTTAATT
PiuApromoter-F	ACTAAAAGTGATTAAAACTTTCACA
PiuApromoter-R	GGCACGTACGAATATACCACATA
RT-qPCR	
rpoB-F	AGTCACGCGAAGTTGAAGGT
rpoB-R	AGCACGCTCAACACGAACTA
piuA-F	TTGCGTACTGTTCCTGGCAT
piuA-R	TCGACCGCAAACATTTCACG
